# Relationships between Gut Microbiota, Metabolome, Body Weight, and Glucose Homeostasis of Obese Dogs Fed with Diets Differing in Prebiotic and Protein Content

**DOI:** 10.3390/microorganisms8040513

**Published:** 2020-04-03

**Authors:** Emmanuelle Apper, Lisa Privet, Bernard Taminiau, Cindy Le Bourgot, Ljubica Svilar, Jean-Charles Martin, Marianne Diez

**Affiliations:** 1 Tereos, Research and Innovation, 77230 Moussy-le-Vieux, France; cindy.lebourgot@tereos.com; 2 MS Nutrition, C2VN, INRA, INSERM, Aix-Marseille University, 13385 Marseille, France; Lisa.privet@gmail.com; 3 Farah Centre, Department of Food Sciences, University of Liege, 4000 Liège, Belgium; Bernard.Taminiau@uliege.be; 4 CRIBIOM, C2VN, INRA, INSERM, Aix-Marseille University, 13385 Marseille, France; Ljubica.SVILAR@univ-amu.fr; 5 BioMeT, C2VN, INRA, INSERM, Aix-Marseille University, 13385 Marseille, France; Jean-charles.MARTIN@univ-amu.fr; 6 Nutrition Unit, Department of Animal Production, Faculty of Veterinary Medicine, University of Liege, 4000 Liège, Belgium; mdiez@uliege.be

**Keywords:** prebiotic, obesity, energy homeostasis, microbiota, metabolome, bile acids, amino acids

## Abstract

Obesity is a major issue in pets and nutritional strategies need to be developed, like promoting greater protein and fiber intake. This study aimed to evaluate the effects of dietary protein levels and prebiotic supplementation on the glucose metabolism and relationships between the gut, microbiota, metabolome, and phenotype of obese dogs. Six obese Beagle dogs received a diet containing 25.6% or 36.9% crude protein, with or without 1% short-chain fructo-oligosaccharide (scFOS) or oligofructose (OF), in a Latin-square study design. Fecal and blood samples were collected for metabolite analysis, untargeted metabolomics, and 16S rRNA amplicon sequencing. A multi-block analysis was performed to build a correlation network to identify relationships between fecal microbiota, metabolome, and phenotypic variables. Diets did not affect energy homeostasis, but scFOS supplementation modulated fecal microbiota composition and induced significant changes of the fecal metabolome. Bile acids and several amino acids were related to glucose homeostasis while specific bacteria gathered in metavariables had a high number of links with phenotypic and metabolomic parameters. It also suggested that fecal aminoadipate and hippurate act as potential markers of glucose homeostasis. This preliminary study provides new insights into the relationships between the gut microbiota, the metabolome, and several phenotypic markers involved in obesity and associated metabolic dysfunctions.

## 1. Introduction

Obesity is a very common nutritional disorder in dogs. Pets are considered to be obese when they weigh 20%–30% more than their ideal body weight [1]. Surveys have revealed a prevalence of canine obesity of 39% in France [2], and about 50% in the UK [3]. Obesity is a major risk factor for many disorders in dogs, such as orthopedic diseases, urinary tract disease, cardiovascular disease, respiratory dysfunction, and hormonal disturbances, and may reduce longevity in the long term. Canine obesity may be associated with the development of insulin resistance, altered lipid profiles, and hypertension [4].

Obesity and insulin resistance have been associated with changes in the composition and function of the gut microbiota. At a species level, a high-fat diet results in decreased SCFA-producing bacteria, such as *Faecalibacterium prausnitzii, Eubacterium rectale*, and *Clostridium coccoides*, while simultaneously inducing inflammatory activity, fat storage, and insulin resistance in mice [5]. In dogs, few studies are available, but one reported that the phylum Actinobacteria and the genus *Roseburia* were significantly more abundant in obese than in lean dogs [6]. Some members of the *Roseburia* genus are recognized as producers of SCFA, with fermentable fibers as the substrate. SCFA-producing bacteria may therefore be more abundant in some obese dogs compared to lean dogs. Obese humans and rodents also seem to have an increased amount of fecal SCFA content compared to lean individuals [7]. SCFAs are thought to benefit the host by improving glucose homeostasis and stimulating enterocyte differentiation, but it should be noted that SCFAs, such as acetate, also serve as an energy source for the host [8]. Another study revealed that the microbial community was less diverse in the obese group compared to the lean group and that bacteria from the phylum Proteobacteria represented the predominant group in the gut microbiota of obese dogs [9]. Another study demonstrated that the family Enterobacteriaceae was overrepresented in dogs with diabetes mellitus [10].

Studies on mice have demonstrated several underlying mechanisms, including host signaling pathways through bacterial lipopolysaccharides (LPSs), bacterial fermentation of carbohydrates and proteins, and bacterial modulation of bile acids, to explain relationships between microbiota, obesity, and insulin resistance [11]. Similarly, in dogs, a modulation of fecal unconjugated bile acids has been observed in diabetes mellitus-suffering dogs compared to healthy dogs [10]. On top of this, increased permeability of the intestinal epithelium could lead to increased absorption of macromolecules from the intestinal content, resulting in a systemic immune response, low-grade inflammation, and altered signaling pathways, influencing glucose and lipid metabolism. Few data are available in obese dogs to highlight the relationships between the gut microbiota, excess body weight, and insulin resistance state [6,9]; however, it is suggested that modulation of the microbiota may modulate glucose metabolism in obese dogs like in other species.

High-protein diets are commonly used to improve glycemic control in obese dogs [12,13,14]. A high-protein diet may stimulate energy expenditure, spare lean mass at the expense of fat mass, and reduce the appetite while increasing satiety, improving the metabolic profile, and releasing gut incretin as GLP-1 [14,15,16]. However, a high-protein diet can alter gut microbiota, notably by causing increased growth of *Clostridium perfringens* while reducing *Bifidobacterium* [17], and increasing malodorous compound production.

Short-chain fructo-oligosaccharides (scFOSs) and oligofructose (OF) are β-fructans defined as prebiotic fibers [18]. Dietary supplementation with scFOSs stimulates the growth of *Bifidobacterium* and certain Lactobacilli in pets [19]. Furthermore, it also improves the peripheral insulin sensitivity index, which is measured by using the euglycemic hyperinsulinemia clamp technique [20] or insulin tolerance test [21,22] in obese or healthy dogs. Thus, regular intake of prebiotic fiber could help improve glucose homeostasis by modulating gut microbiota. Furthermore, use of β-fructans may alleviate the potential negative effects observed with a high-protein diet, notably the alteration of some gut bacteria and the production of malodorous compounds. Thus, prebiotics may have synergistic effects with high-protein diets used in marketed foods for obese dogs.

Few data are available to describe the relationships between diet, microbiota, and modification of the glucose metabolism in obese dogs. The objectives of this study were firstly to measure blood glycemia and insulinemia and to describe the gut microbiota of obese dogs fed diets differing in protein levels and β-fructan supplementation, and secondly to establish possible relationships between microbiota and phenotypic parameters using non-invasive methods. Such data could help (1) to understand the mechanisms involved by which proteins and prebiotics mitigate glycemic control, and (2) to determine the specific metabolites related to an overweight and energetic status in dogs.

## 2. Materials and Methods

The experimental protocol was approved the 4th of July 2013 by the Ethics and Scientific Integrity Council (C.E.I.S.) of the University of Liege (Belgium) and the agreement was registered under the number No. 1463.

### 2.1. Dogs and Feeding

Six overweight adult Beagle dogs that had been overweight for 12 months prior to the experiment (neutered females; age = 8 years, mean BW = 13.56 ± 1.36 kg, BCS ± 8/9 [23]) were included in the study. Dogs were pair-housed in an outdoor paddock (3.85 × 4 m) during the whole experiment, except for 1 week per period where they were placed in metabolic cages (1 × 1.2 m) for a total of 5 days for feces collection. All dogs were vaccinated and wormed before the study. Animals were healthy according to the physical examination and biochemistry tests. Dogs were fed once a day at 9 a.m. and had unlimited access to fresh water.

The dogs were divided into two groups of three and fed with one of two dry foods (normal protein, 25.6% (NP) and high protein, 36.9% (HP)) formulated for the experiment to meet maintenance requirements (Table 1) based on NRC [24]. Protein was included at the expense of carbohydrates. The energy contribution from protein, fat, and carbohydrates was 25%, 28%, and 47% in NP and 36%, 28%, and 36% in HP. Amounts of food were adjusted weekly to maintain a stable BW that was assessed weekly.

### 2.2. Experimental Design

The experiment consisted of two 3 × 3 complete Latin squares. Each Latin square period lasted for 17 weeks, followed by 3 weeks of wash-out. Each Latin square period corresponded to 1 diet (NP or HP) and was divided into 3 sub-periods of 5 weeks separated by 1 week’s wash-out between each sub-period. Each dog received either the control diet (CTRL), the diet supplemented with 1% DM oligofructose (OF, Orafti^®^OLF, Beneo, Oreye, Belgium), or with 1% DM scFOS (scFOS: degree of polymerization, 3–5, Profeed^®^, Tereos, Marckolsheim, France). The scFOS or OF supplement was top-dressed on the food, immediately before giving the meal. Finally, each dog tested each protein level and each prebiotic supplementation during the whole 37-week trial.

### 2.3. Samples

Dogs were weighed during the last week of each sub-period. They were also placed in metabolic cages during the last week of each sub-period.

Feces samples were collected during the last 5 days to calculate the wet and dry matter output. The feces were kept at 4 °C until the end of the week. Feces samples for analysis of the microbiota were taken directly in the rectum, using a sterile plastic probe (CH18), and then brought immediately to the lab for total DNA extraction. A fresh sample of 20 g of feces was collected the last day, and after homogenization of the output, it was stored at −80 °C for the metabolomic analysis. Fecal water to perform metabolomic analysis was prepared as follows: After thawing, 1 g of feces was blended with 1 mL of deionized water and placed into a vortex for 30 s. Samples were then put in a bath sonicator in iced water for 2 min, and then placed in the vortex for 30 s. Fecal samples were centrifuged at 171,500× *g* for 30 min at 4 °C and the supernatant was collected and stored at −80 °C until further analysis.

Blood samples were collected by venipuncture into the cephalic vein after a 24-h food-free period at the end of each Latin square period. Fasting glucose and haptoglobin were measured the same day. The samples were taken in tubes stored at 4 °C and centrifuged (3000× *g*; 20 min). Plasma was stored at −20 °C for fasting cholesterol and insulin analysis and at −80 °C for metabolomic analysis.

### 2.4. Measures

#### 2.4.1. Feces Characteristics

Collected feces were dried in a 70 °C oven until a stable weight was reached at 103 °C to determine the dry matter output. During the last 5 days of each period, feces were scored daily using the Waltham feces score system [25]. The number of stools per day was also recorded.

#### 2.4.2. Blood Parameters

Glycemia was controlled by using a glucometer (Accu-chek Active, Roche Diagnostics). Plasma insulin was measured using radioimmuno kits (Biosource INS-IRMA, Biosource Europe). Total plasma cholesterol concentrations were assessed by colorimetric methods (kit Ecoline S+, Diagnostics System). The homeostasis model of insulin resistance (HOMA-IR) was also calculated from basal glycemia (G0; mmol/L) and basal insulinemia (I0; mU/L), with both using the Oxford calculator and the following HOMA-IR = (G0 × I0)/22.5 [26]. An assay was developed at the Biochemistry Unit laboratory at the Faculty of Veterinary Medicine in Liege in order to measure the plasma canine haptoglobin concentration. This is a photometric method that measures the peroxidase activity of the haptoglobin–cyanmethaemoglobin complexes, as described elsewhere [27].

#### 2.4.3. Fecal Concentrations of SCFA and BCFA

All standard products were acquired from Sigma Aldrich, France. Acetic, propionic, butyric, isobutyric, valeric, and isovaleric acids were quantified in fecal water samples using gas chromatography-mass spectrometry (GC-MS). For this purpose, fatty acids were derivatized by propyl chloroformate (PCF) to enhance their mass and to provide good derivatization efficiency. A concentration of 6.25 µg/mL acetic acid-d4 in water was used as the labelled internal standard. A combined solution of six acids was prepared in water at a 1 mg/mL concentration and a serial dilution was performed to obtain nine solutions with final concentrations of 3.91, 7.8, 15.6, 31.3, 62.5, 125, 250, 500, and 1000 µg/mL. To produce a calibration curve, calibration points were prepared in triplicates. Briefly, 2 µL of internal standard solution was added to 200 µL of each solution combination or 200 µL of fecal water. Then, 333 µL of 0.005 M NaOH solution and 333 µL of n-propanol (PrOH)/Pyridine, (3/2, *v*/*v*) were added. A quantity of 66 µL of PCF was added and gently stirred until the complete elimination of carbon dioxide. After the addition of 300 µL of hexane, the extract was vortexed, centrifuged and the upper phase was isolated. This step was repeated, and upper phases were combined and dried using 10 mg of Na2SO4. Dried extracts were transferred to vials and analyzed by GC-MS. The Agilent 5890 GC system coupled to an MS 5973N mass detector was used to separate and detect the derivatives. The Agilent HP-5MS (30 m × 0.25 mm × 0.25 µm) column was used to separate 1 µL of derivative molecules injected in split mode (10:1 split ratio). Helium was used as the carrier gas at a constant flow of 36 cm/s. Solvent delay was set to 2 min. Initial temperature was set at 50 °C, with a temperature rise to 70 °C after 2 min, to 85 °C after the next 5 min, to 115 °C after the next 5 min, and to 290 °C after 11 min, maintained for 5 min, before increasing to 325 °C for 6.75 min and maintaining for 5 min. Inlet, source, and quadrupole temperature were maintained at 300, 230, and 150 °C, respectively. Electron ionization was achieved using −70 eV electron energy and mass spectra were acquired in the 40–600 *m*/*z* window. Calibration curves were created using the ratio between the peak areas of the SCFA and internal standard. The ratios were plotted on the *y*-axis and analyte concentration in µg/mL on the *x*-axis. A regression curve was constructed using the y = ax equation and covering the concentration range from 1.3 to 333 µg/mL.

#### 2.4.4. Microbiota Analysis

Feces samples from the rectum were used to analyze microbiota. PCR-amplification of the V1-V3 region of the 16S rDNA and library preparation were briefly performed with the following primers (with Illumina overhand adapters), forward (5′-GAGAGTTTGATYMTGGCTCAG-3′) and reverse (5′-ACCGCGGCTGCTGGCAC-3′). Each PCR product was purified using the Agencourt AMPure XP beads kit (Beckman Coulter, Pasadena, USA) and submitted to PCR a second time for indexing, using the Nextera XT index primers 1 and 2. After purification, the PCR products were quantified using the Quant-IT PicoGreen (ThermoFisher Scientific, Waltham, USA) and diluted to 10 ngµL-1. Final quantification, by qPCR, of each sample in the library was performed using the KAPA SYBR^®^ FAST qPCR Kit (KapaBiosystems, Wilmington, USA) before normalization, pooling, and sequencing on an MiSeq sequencer using v3 reagents (ILLUMINA, USA). Sequence read processing was used as previously described using MOTHUR software package v1.35, Pyronoise algorithm, and UCHIME algorithm for alignment and clustering, denoising, and chimera detection, respectively. 16S reference alignment and taxonomical assignation were based on the SILVA database (v1.19) of full-length 16S rDNA sequences [28].

#### 2.4.5. Metabolomic Analysis

Plasma and fecal samples were analyzed using the non-targeted metabolomic approach. GC-MS and LC-HRMS were used for this purpose.

For plasma analysis, 100 µL of sample was protein precipitated with 400 µL cold methanol (−20 °C). After 1 h of incubation at −20 °C, 1 min of vigorous shaking (vortex), and 15 min of centrifugation at 4 °C and 13,552× *g*, the supernatants were divided in two parts. The first part, used for the GC-MS analysis, was evaporated under a nitrogen flow, while the second was filtered through a 10-kDa filter (VWR) and then evaporated under gentle nitrogen flow. For the GC-MS analysis, tripthophan-d5 and cholesterol-d4 were added to a final concentration of dry extracts at 1 µg/mL. Then, 25 µL of methoxamine hydrochloride (Sigma Aldrich, France) in pyridine was added to the dried extract and incubated for 15 min at 80 °C. Then, 25 µL of N-Methyl-N-(trimethylsilyl) trifluoroacetamide (MSTFA) (Sigma Aldrich, France) was added to the extract and the incubation was repeated. Finally, 5 µL of alkane mix solution was added for the retention indices, and the samples were transferred to glass vials. Pooled samples were prepared by assembling 10 µL of each sample and interspacing every 5 randomized samples. For the LC-MS analysis, dry extracts were dissolved in 125 µL of water/acetonitrile (90/10, *v*/*v*). Pooled samples were prepared by assembling 20 µL of each sample and injecting it into LC-MS every 5 randomized samples.

Fecal water samples were prepared as follows: 90 µL of fecal water sample were diluted in 310 µL of 50 mM NH_4_·HCO_3_ solution and vortexed for 1 min. Then, 1.6 mL of cold methanol (−20 °C) were added to the sample, vortexed, and then centrifuged for 15 min at 4 °C and 13,552 g. Then, 150 µL of supernatant were filtered through the 10-kDa centrifugal filters (VWR International, France), evaporated, and reconstituted in 300 µL of water/acetonitrile (90/10, *v*/*v*) for the LC-MS analysis. Another 150 µL of supernatant were evaporated and derivatized as described above for the GC-MS analysis. Pooled samples were prepared by assembling 10 µL of each sample and injecting this into GC-MS every 5 randomized samples.

For the GC-MS analysis, an Agilent HP-5MS (30 m × 0.25 mm × 0.25 µm) column was used to separate 1 µL of derivative molecules injected in split mode (10:1 split ratio). Helium was used as the carrier gas at the constant flow of 36 cm/s. Solvent delay was set to 2 min. Initial temperature was set at 70 °C and maintained for 2 min. Then, over 27 min, the temperature was raised to 325 °C at a rate of 10 °C/min and maintained for 10 min at 325 °C. The inlet, source, and quadrupole temperature were maintained at 300, 230, and 50 °C, respectively. Electron ionization was achieved using −70 eV electron energy and mass spectra were acquired in the 70–600 *m*/*z* window. Peak areas were integrated using the Total Chrom 6.3.1 (Perkin Elmer) program and then normalized using the labelled standards tripthophan-d5 and cholesterol-d4. A total of 70 metabolites were identified and integrated from GC-MS analysis of plasma samples and 75 in fecal water samples.

For the LC-MS analysis, chromatographic separation was carried out on a Dionex UltiMate 3000 (Thermo Fisher Scientific) consisting of a rapid separation pump (RS) (LPG-3400 RS), an autosampler (WPS- 3000 TRS), and a column compartment (TCC-3000 RS) all operated by Chromeleon 6.8 software. A reverse-phase Nucleodur C18 Isis (150 mm × 2 mm × 1.8 µm) (Macherey-Nagel, France) and Hypersil Gold (100 mm × 2.1 mm × 1.9 µm) (Thermo Scientific, France) column were used for compound separation in the plasma and fecal samples, respectively. Here, 0.1% formic acid solutions in water and acetonitrile were used as solvents A and B, respectively, as mobile phases. The elution gradient for plasma analysis was as follows: Solvent B was maintained at 5% during the first minute. A linear gradient then raised solvent B to 50%, maintained it for 2 min, before raising it again to 97% of B for the next 6 min. Then, 97% of solvent B was held in isocratic conditions for 2 min, after which initial conditions were restored, and the column re-equilibrated for 4 min. The elution gradient for fecal analysis was as follows: 0% of solvent B was kept in isocratic conditions for 1 min, after which a linear gradient raised solvent B to 100% in 10 min. After 2 min with B at 100%, the initial conditions were restored and equilibrated for 2 min. Full MS spectra were acquired on a Q-Exactive Plus mass spectrometer (Thermo Fisher Scientific, Bremen, Germany) with a Heated Electrospray Ionization (H-ESI II) probe working in the switching polarity mode. The spray voltage was kept at 3.5 kV; the capillary and auxiliary temperature were 320 and 310 °C, respectively; the sheath and auxiliary gas flow rate were 30 and 8 arbitrary units, respectively; and the S-lens RF was kept at 55 V. Spectra were acquired using a resolving power of 70,000 full width at half maximum (FWHM) for the theoretical *m*/*z* 200, and the injection time to C-trap was set at 250 ms. Spectra were acquired in the mass range 8–1000 *m*/*z*. Thermo Xcalibur 3.0.63 software was used for the instrument setup and control of the LC-MS system during acquisition. The Tune Q Exactive Plus 2.5 application was used to control the mass spectrometer directly. After the acquisition of the raw data and manual inspection of the spectra, automatic peak detection and integration were performed in the XCMS package. A total of 2596 ion features were obtained from LC-MS analysis of plasma samples and 916 in fecal water samples after post-processing.

### 2.5. Statistical Analysis

#### 2.5.1. Univariate Analysis

A linear model adjusted on dog and period effects was used to measure the effect of dietary protein level (NP vs. HP) and supplementation (CTRL, scFOS, and OF) on body weight and excess body weight excess, and blood and fecal variables (R software). Interactions between protein and prebiotics were tested and then removed as they were not significant. Differences were considered significant for a *p*-value < 0.05, and trends were notified for a *p*-value < 0.10.

#### 2.5.2. Amplicon Sequencing

To analyze these data independently, subsample datasets were collected and used to evaluate ecological indicators, richness estimation (Chao1 estimator), microbial biodiversity (reciprocal Simpson index), and population evenness (derived from the Simpson index) using MOTHUR [29]. Population structure and community membership were assessed using MOTHUR, with the distance matrix based on the Bray–Curtis dissimilarity index (a measure of community structure, which considers shared OTUs and their relative abundances; [30]). Ordination analysis and 3D plots were performed using the Vegan, Vegan3d, and rgl packages in R (Dixon, Philip). Non-metric dimensional scaling based on the Bray–Curtis dissimilarity matrix was applied to visualize the biodiversity between the groups. An AMOVA test was performed to assess the diversity clustering of treatment groups with the Bray–Curtis matrix using MOTHUR [31]. Statistical differences between bacterial biodiversity, richness, and evenness were assessed using ANOVA corrected for multi-testing (Benjamini–Hochberg using PRISM 6 (Graphpad Software)). Differences were considered significant for a *p*-value of less than 0.05. Statistical differences in population abundance between treatment groups were assessed using ANOVA, corrected for multi-testing (Benjamini–Hochberg false discovery rate) using the STAMP software [32]. Statistical differences between treatment groups for specific bacterial populations were assessed by two-way ANOVA and Tukey–Kramer post hoc test using PRISM 6 (Graphpad Software). Differences were considered significant for a *p*-value of less than 0.05. All the biosample raw reads have been deposited at the National Centre for Biotechnology Information (NCBI).

#### 2.5.3. Global Relationships between Microbiota Composition and Activity, and Phenotypic Variables

**Figure 1:**
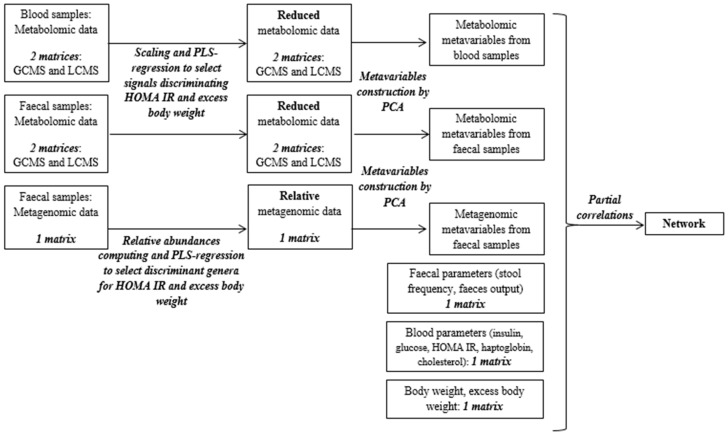
Workflow of the various statistical analyses performed to produce the correlation network.

*Data preparation.* The workflow used to build the correlations network is shown in Figure 1. The 8 following biological data matrices were used: Metabolomic data acquired by LC-MS and GC-MS analysis on blood samples (*n* = 2), metabolomic data acquired by LC-MS and GC-MS analysis on fecal samples (*n* = 2), and amplicon sequencing data acquired by analysis of the fecal microbiota (*n* = 1), fecal composition data (*n* = 1), blood parameters (insulin, glucose, cholesterol, haptoglobin, HOMA IR; *n* = 1), and body weight and excess body weight percentage (*n* = 1). After logarithmic transformation and pareto scaling for the LC-MS data and relative abundance computing for the metagenomic data, a PLS-Regression was run to select variables linked to the HOMA IR, body weight, and excess body weight from each of these large matrices (on all matrices except phenotypic data; Figure 1) using the SIMCA software (Umetrics, Umea, Sweden). A principal component analysis was then run on the reduced matrices to extract latent variables containing key information (using the FactoMineR package from the R software). These latent variables were called metavariables.

*Statistical analysis*. A grouped and weighted factorial analysis (multiple factorial analysis) was run on the multi-block data made up of the metavariables based on metabolomic and amplicon sequencing data, and on all phenotypic variables to visualize correlations between variables, groups of variables, and individual dispersion (using the FactoMineR package from the R software). Partial correlations between all variables and metavariables were computed using the GeneNet package from R software, as described by Opgen-Rhein and Strimmer [33,34] and Schäfer and Strimmer [35]. A correlations network was computed to represent pair-wise relationships between the different kinds of data using Cytoscape software. Only edges showing correlations different from 0 with a *p*-value under 0.05 were drawn. For each metavariable representing the information contained in a matrix of data, the correlations between this metavariable and the original variables of this matrix were estimated to facilitate interpretation of the correlations network. A dog- and period-adjusted linear model was used to measure the effect of dietary protein level (NP vs. HP) and prebiotic supplementation (CTRL, scFOS, and OF) on the different metavariables. Interactions between protein and prebiotics were tested and then removed as they were not significant. Differences were considered significant for a *p*-value < 0.05, and trends were notified for *p*-value < 0.10.

## 3. Results

### 3.1. Effects of Dietary Protein Level and Prebiotic Supplementation on Host Phenotypic Parameters

No significant interaction was found between prebiotics and proteins. Body weight averaged at 13.4 kg and did not differ between groups. Excess body weight percentage was higher than 50%, with no significant effect from the diet. Modifying dietary protein levels or supplementing the diet with prebiotics did not affect body weight parameters and fasting blood insulin, glucose, haptoglobin, and cholesterol levels (Table 2). However, fasting insulin numerically decreased by 8.2% with the HP diet and by 14.1% with the scFOS diet (Table 2).

Fecal parameters remained unchanged regardless of the dietary protein level (Table 3). The scFOS group had higher fecal concentrations of butyrate compared to OF and CTRL (*p* = 0.039; Table 3). It should be noted that there was also a tendency toward a decreased amount of total SCFA in the feces of dogs supplemented with prebiotics (*p* = 0.109), especially with the scFOS vs. CTRL group. This reduction of total SCFA production seems to be particularly due to the decrease of acetate with scFOS intake.

### 3.2. Effects of Dietary Protein Level and Prebiotic Supplementation on Fecal Microbiota

**Figure 2:**
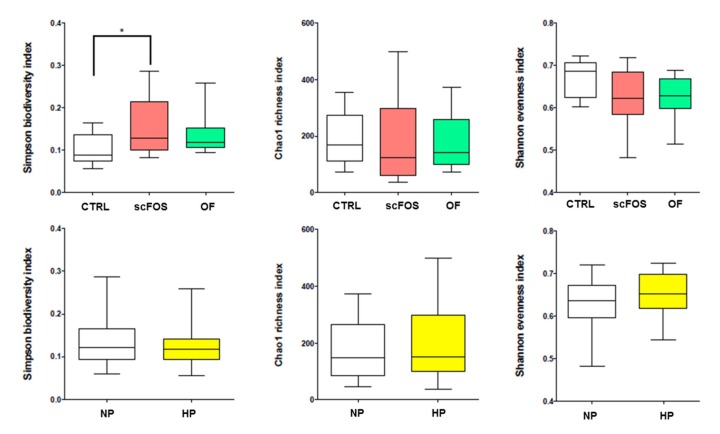
Effects of prebiotic supplementation and dietary protein level on ecological indicators of fecal microbiota in obese dogs. CTRL: control; OF: oligofructose; scFOS: short-chain fructo-oligosaccharides; NP: normal protein; HP: high protein. * *p* < 0.05.

Fecal microbiota ecological indicators were not modified by the level of dietary proteins, while the diversity index was significantly higher with the scFOS than with the CTRL diet (Figure 2).

**Figure 3:**
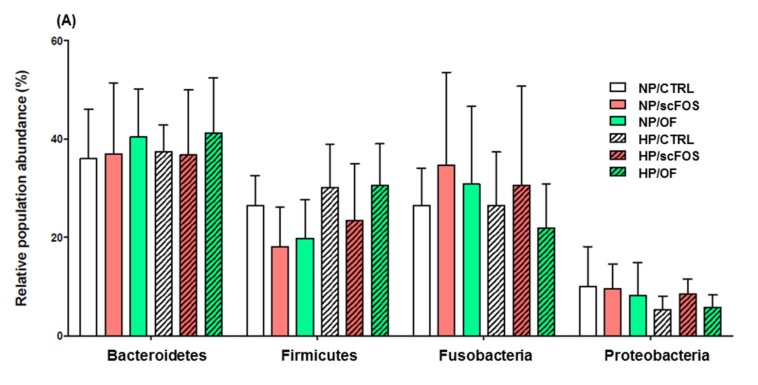

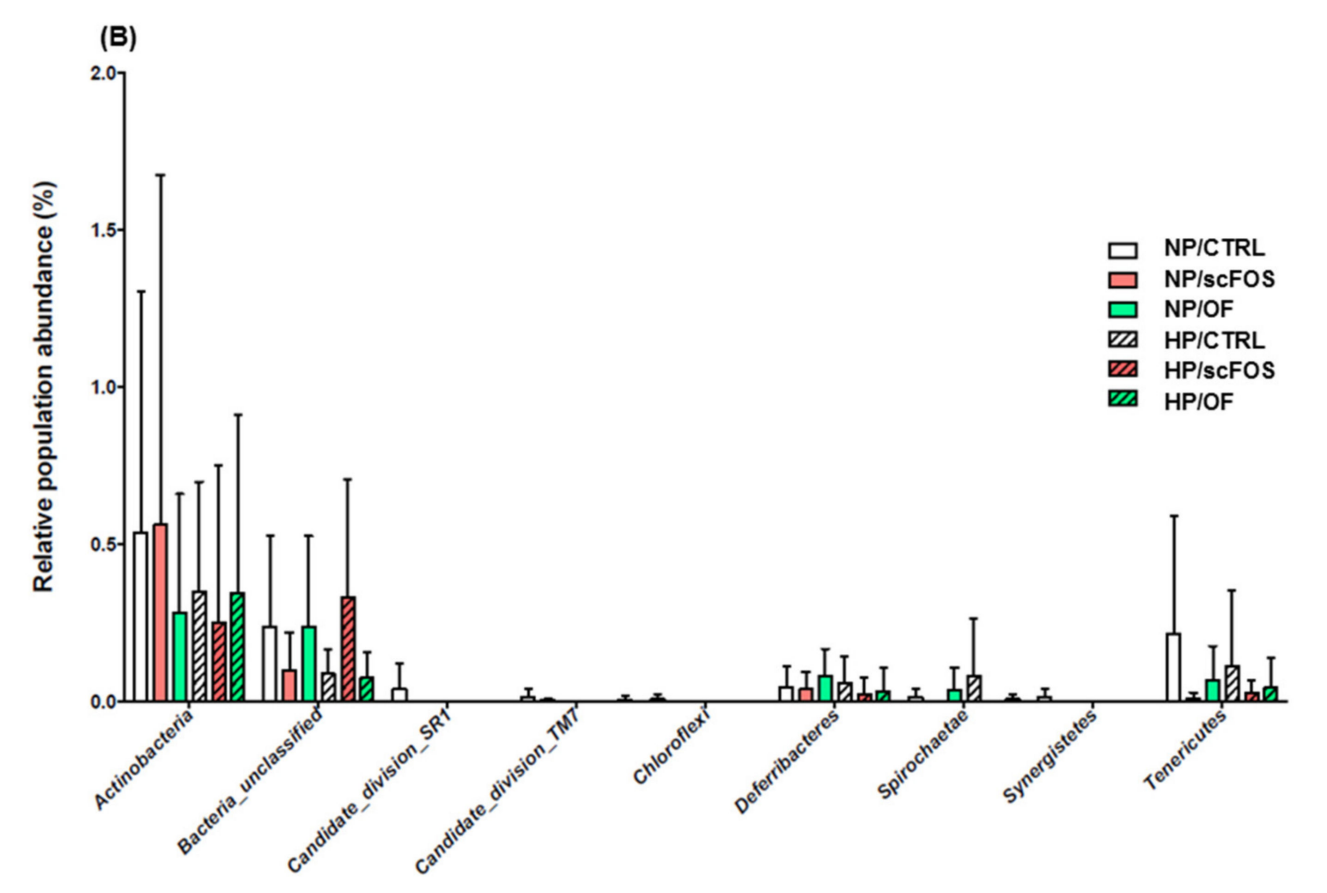
Effects of prebiotic supplementation and dietary protein level on major (**A**) and minor (**B**) phyla found in the fecal microbiota of obese dogs. CTRL: control; OF: oligofructose; scFOS: short-chain fructo-oligosaccharides; NP: normal protein; HP: high protein.

Regardless of dietary treatment, Bacteroidetes (35%–40% of all sequences), Fusobacteria (23%–38% of all sequences), and Firmicutes (18%–25% of all sequences) were the predominant bacterial phylum in dog feces. Proteobacteria (less than 10% of all sequences) was also present. Tenericutes and Actinobacteria were detected at less than 0.5% in all sequences. The proportions of phyla were not significantly affected by the protein level or by prebiotic supplementation (Figure 3).

**Figure 4:**
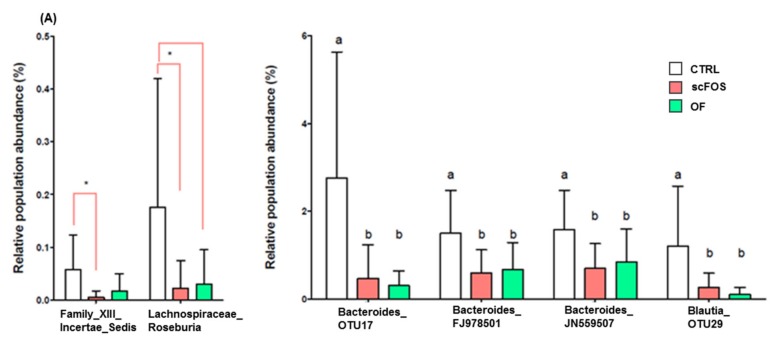

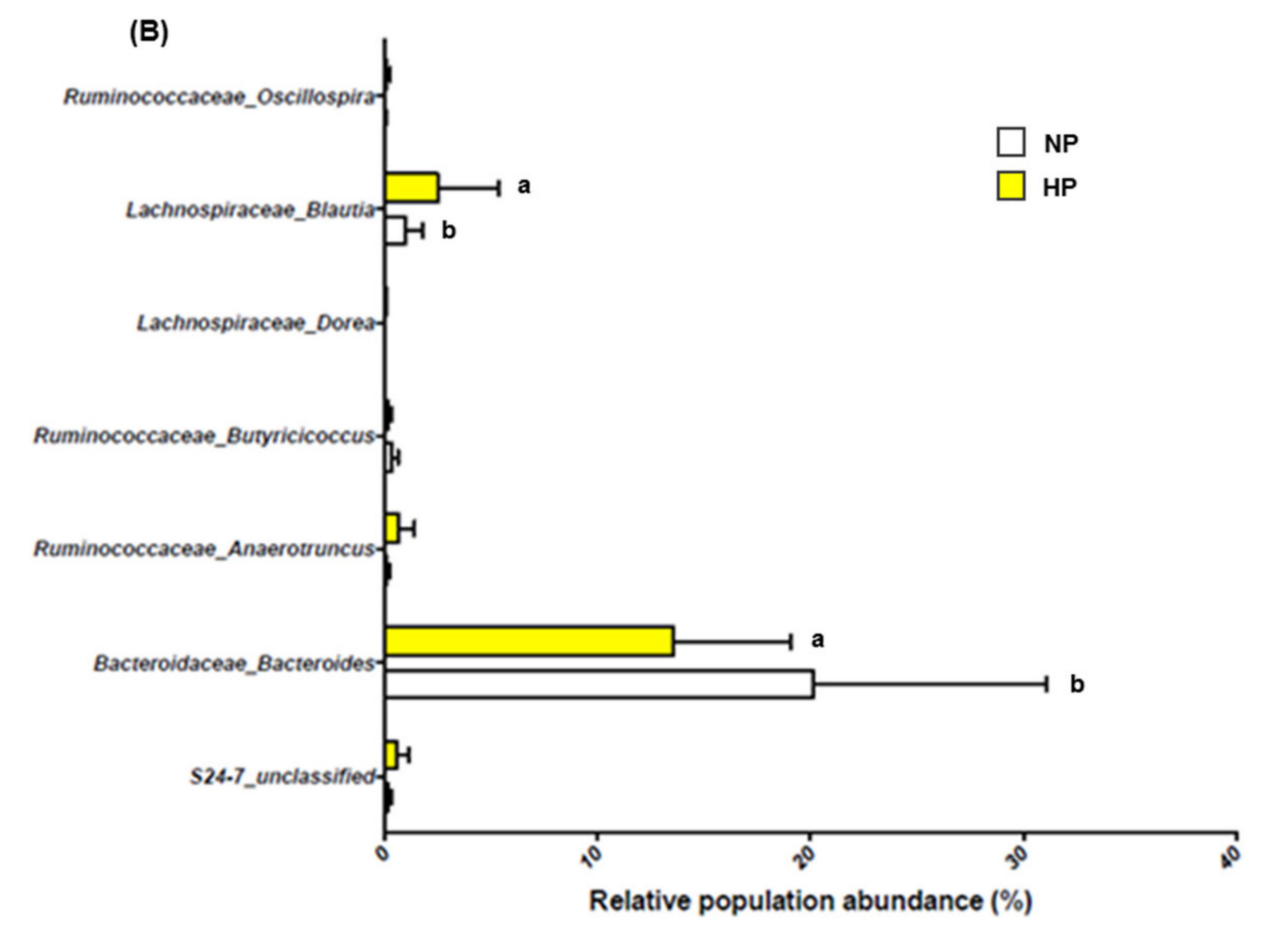
Effects of prebiotic supplementation (**A**) and dietary protein level (**B**) on the bacterial genus and species of fecal microbiota in obese dogs. CTRL: control; OF: oligofructose; scFOS: short-chain fructo-oligosaccharides; NP: normal protein; HP: high protein. a, b: Means within a raw with different superscripts differ significantly: *p* < 0.05. * *p* < 0.05.

Significant differences between dietary protein levels were observed at the genus level, as the proportion of *Lachnospiraceae Blautia* was higher, while that of *Bacteroidaceae bacteroides* was lower with the HP diet than with the NP diet (Figure 4A). Prebiotic supplementation resulted in a reduced proportion of Clostridiales Family XIII *Incertae Sedis* (scFOS only) and *Lachnospiraceae Roseburia* (scFOS and OF; Figure 4B). Prebiotic supplementation significantly reduced the proportion of different unclassified *Bacteroides*, and *Blautia* (Figure 4B).

### 3.3. Relationships between Gut Microbiota and Host Metabolism Parameters

Table 4 reports the means, medians, minimum, maximum, and standard deviation values for the parameters we used in the correlations network and shows the inter-individual variability that made it possible to perform our analysis.

#### 3.3.1. Metabolomic Metavariables

Based on a preliminary PLS analysis, we selected 75 signals related to fecal water metabolites measured by LC-MS (R2 = 74.2%, VIP > 1.80, Q2 = 50.2%, and *p* = 0.001), 30 by GC-MS (R2 = 86.9%, VIP > 1.00, Q2 = 54.9%, and *p* = 0.001), and 53 signals related to plasma metabolites (R2 = 83.4%, VIP > 1.80, Q2 = 48.4%, and *p* = 0.048). From these selected models, principal component analysis was used to build four fecal water metavariables called FaecalGD1, FaecalGD2, FaecalLD1, and FaecalLD2 (Table 5) and one plasma metavariable called PlasmaLD2 (Table 5) in relation to either body weight or HOMA IR. The name of each metavariable was chosen according to the following parameters: Fecal or plasma corresponding to the biologic matrix; G or L corresponding to the method used to identify metabolites, namely GC-MS (G) and LC-MS (L); and D1, D2 corresponding to the dimension obtained according to the principal component analysis in which the metavariable was significant.

FaecalLD1 metavariable gathered discriminant metabolites related to bile acids, namely cholic, ursocholic, taurocholic, and deoxycholic acids, and to amino acids, including phenylalanine, tryptophan, and norleucine. Discriminant metabolites of the faecalGD1 metavariable were amino-acids, namely L-alanine, L-valine, L-proline, L-threonine, L-phenylalanine, and cadaverine, produced by the microbial degradation of lysine.

FaecalLD2 was characterized by different types of metabolites: Hexanoylcarnitine (lipid and amino-acid metabolism); limonene, which is involved in lipid metabolism in relation to primary bile acids; two metabolites derived from phenylalanine and lysine metabolism, respectively, hippurate and aminoadipate; and acetylcholine, a neurotransmitter. FaecalGD2 was characterized by the following metabolites: Phenylpropanoate, involved in aromatic compound (including phenylalanine) degradation and in polyphenol metabolism, and D-fructose. PlasmaLD2 metavariable was associated with a leucine-asparagine dipeptide, hydroxyproline, derived from proline, a marker of bone resorption, muscle degradation, and stress; diaminoheptanediopate, derived from lysine and specific to certain cell walls of gram negative bacteria, and acylcarnitine produced from lysine and methionine and involved in fatty acid catabolism.

ANOVA revealed that the composite score of the metavariable PlasmaLD2 tended to be lower with the HP diet than with the NP diet (*p* = 0.09). Metavariable FaecalGD2 was significantly impacted by scFOS supplementation (*p* = 0.013), the value of the composite score being higher with scFOS than with OF and CTRL (Table 6).

#### 3.3.2. Metagenomic Metavariables

Based on preliminary PLS analysis, we selected different bacterial genera related to body weight and excess body weight (*n* = 34 signals; R2 = 72.9%, VIP > 1.00, Q2 = 27.5% and *p* = 0.017) and others related to HOMA IR (*n* = 14 signals; R2 = 65%, VIP > 1.45, Q2 = 18.7%, and *p* = 0.05). From these genera, three separate metavariables were built (GenusD1; D2 and D3; Table 7). Bacteria from the genera *Lactobacillus*, *Bacteroidales unclassified*, *Allobaculum*, *Lactobacillales* unclassified, *Escherichia-Shigella*, *Parabacteroides*, and *Campylobacter* contributed to the metavariable GenusD1. *Parabacteroides*, *Phascolarctobacterium*, *Campylobacter*, *Gammaproteobacteria* unclassified, *Fusobacterium*, *Wolinella*, and *Brachyspira* contributed to the metavariable GenusD2. GenusD3 was characterized by the following discriminant genera: *Enterococcus*, *Escherichia-Shigella*, *Allobaculum*, *Allisonella*, *Campylobacter*, *Clostridium sensu strictu*, and *Anaeroplasma*. Neither protein level nor prebiotic supplementation significantly affected these metavariables.

#### 3.3.3. Global Network

**Figure 5:**
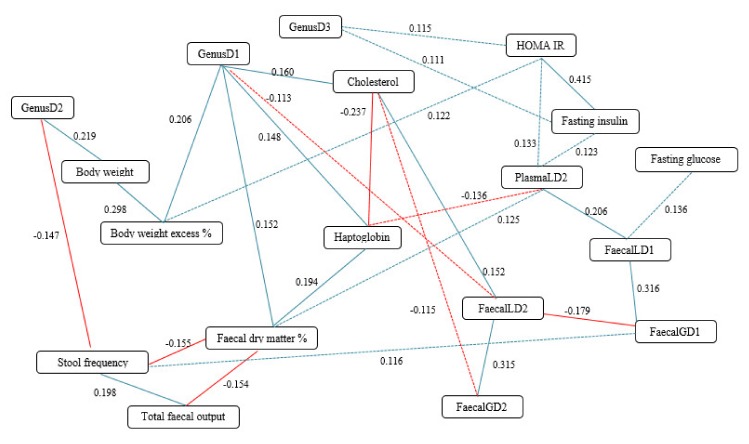
Diagram of the different partial correlations resulting between phenotypic parameters and metagenomic and metabolomic metavariables in obese dogs. In blue: positive partial correlation; in red: negative partial correlation. Solid line: *p* value < 0.01 and Q value < 0.05; Dotted line: *p* value < 0.05 and Q value < 0.15.

As expected, an excess body weight percentage was correlated with the body weight of the dogs (R2 = 0.298: *p* < 0.05, Figure 5; Table 8). Interestingly, body weight was also strongly and positively correlated to the metavariable GenusD2 (R2 = 0.219: *p* < 0.05, Figure 5; Table 8), while excess body weight percentage was significantly and positively correlated with the metavariable GenusD1 (R2 = 0.206: *p* < 0.05, Figure 5; Table 8). The last metavariable was also correlated to the blood cholesterol concentration (R2 = 0.160: *p* < 0.05, Figure 5; Table 8) and haptoglobin concentration (R2 = 0.148: *p* = 0.005, Figure 5; Table 8), and to the percentage of fecal dry matter (R2 = 0.152: *p* < 0.004, Figure 5; Table 8). GenusD2 was negatively correlated with stool frequency (R2 = −0.147, *p* = 0.006). Fecal dry matter was positively correlated with the blood haptoglobin concentration (R2 = 0.194, *p* < 0.001).

A strong correlation was found between fasting insulin and HOMA IR (R2 = 0.415, *p* < 0.0001). We observed a positive correlation between fasting insulin and GenusD3 (R2 = 0.111, *p* = 0.04), between HOMA IR and excess body weight (R2 = 0.122, *p* = 0.004), and between fasting insulin and the plasmaLD2 (R2 = 0.123, *p* = 0.02). PlasmaLD2 was significantly and positively related to FaecalLD1 (R2 = 0.206, *p* < 0.0001) and to HOMA IR (R2 = 0.133, *p* = 0.01).

Overall, all fecal metavariables were strongly inter-related (Figure 5, Table 8). FaecalLD2 was also positively correlated with blood cholesterol (R2 = −0.115, *p* = 0.03), and negatively related to GenusD1 (R2 = −0.113, *p* = 0.034). Stool frequency was negatively related to GenusD2 (R2 = −0.147, *p* = 0.006) and fecal dry matter (R2 = −0.155, *p* = 0.003) and positively to total fecal output (R2 = 0.198, *p* = 0.0002).

Interestingly, the GenusD1 node showed the greatest number of links and connected different regions of the network together, corresponding to various kinds of data. It thus appears to be an important multiscale regulatory hub.

## 4. Discussion

The primary objective of our study was to measure the effects of prebiotic supplementation on glucose homeostasis in obese dogs fed diets containing different amounts of protein. The secondary objective was to better understand the relationships between fecal microbiota and the phenotypic parameters of obese dogs using non-invasive methods.

As expected, all obese dogs exhibited an excess body weight percentage of over 50 (Table 2 and Table 4). Furthermore, fasting insulin and glucose reached the same average levels as those observed by Respondek et al. [20] in obese and insulin-resistant dogs even if we had huge variability. Blood cholesterol was high, just under 7.76 mmol/L, typical of hyperlipidemia [36]. Similarly, blood haptoglobin concentrations were elevated, similar or even higher than the values found by Ricci et al. [37]. Despite the significant variability observed, we considered that our dogs were obese.

Dietary protein content did not impact the measured parameters while we hypothesized that it could have an effect on the glucose homeostasis of our obese dogs. However, this lack of effect can be related to the fact that dogs were fed to maintain a constant body weight during all the study to avoid a confounded effect between the loss of weight and the diet on glucose homeostasis parameters. The different papers related to this topic in dogs described an improvement of glucose homeostasis with a high-protein diet related to a weight loss [12,13,14]. However, the fact that the dietary protein tended to impact one of the plasma metavariables (see below) suggests that they can mitigate glucose homeostasis.

Prebiotic supplementation did not modify glucose homeostasis-related parameters, although it resulted in numerically decreased fasting insulin (−14.1% and −17.0% with scFOS compared to CTRL and OF, respectively). The small number of dogs and the high inter-individual variability might have contributed to the lack of significance of these effects. Furthermore, the effects of scFOS and OF supplementation on fasting glucose and insulin are inconsistent in different animal models [20,38,39,40] and also in humans [41]. The results of these studies suggest that fasting parameters are poor markers for evaluating the effect of prebiotic supplementation on glucose homeostasis. Such findings have been confirmed by a study using dogs to compare the predictive quality of fasting parameters, including HOMA-IR, with data obtained from high-quality predictive models [42]. In the current study, the use of dynamic models would have been more efficient; however, we wanted to avoid invasive methods and to use “easy-to-sample” markers to evaluate the efficiency of the diet on glucose homeostasis.

Supplementation with prebiotics, and especially scFOS, had positive impacts on the gut microbiota composition and fermentative activity. Supplementation with scFOS primarily increased the Simpson diversity index. Park et al. [9] observed a decrease in the microbial community diversity in obese dogs compared to lean dogs. Similarly, different studies showed that dysbiosis and poor species diversity were associated with obesity in rodents and humans [43,44]. A recent study in humans reported that both the Shannon and Simpson indexes were reduced in a group of women showing weight gain compared to a group of women showing weight loss, and that dietary fiber intake was positively correlated with these indexes [45]. Secondly, prebiotic supplementation impacted the microbiota composition and notably affected bacteria that have been associated with obesity. Indeed, relative proportions of *Lachnospiraceae Roseburia, Blautia*, and *Clostridiales Family XIII Incertae Sedis* (scFOS only) were decreased following prebiotic intake. Interestingly, the genus *Roseburia* has been reported to be present in higher proportions in obese dogs [6] while the Lachnospiraceae family increased in mice fed a high-fat diet and decreased in weight-reduced mice [46]. *Clostridiales Family XIII Incertae Sedis* have been found in a higher relative abundance in human subjects with constipation-predominant intestinal bowel disease [47]. Furthermore, some species of *Bacteroides* unclassified have also been reduced with prebiotic intake. A dominance of *Bacteroides* has been observed among obese individuals, with a demonstrated positive correlation between *Bacteroides* distribution and body mass index [48]. *Bacteroides* were also earlier correlated to weight gain and diabetes in pregnant women [49] and in diabetes-prone rats [50]. Interestingly, the genus *Bacteroides* was discriminant in our metavariable in relation to an excess body weight percentage.

Fecal butyrate concentration was significantly higher with scFOS than with CTRL and OF supplementation. Butyrate is the main energy supply for colonocytes, and non-production of butyrate could modify the structure of the intestinal epithelium, leading to increased intestinal permeability and the passage of molecules from the intestinal lumen to the bloodstream [51], including lipopolysaccharides, which are involved in low-grade inflammation [5]. Furthermore, butyrate can regulate the levels of GLP-1, which enhances glucose-dependent insulin secretion by pancreatic β-cells. In addition, it should be noted that the prebiotic tended to decrease the amount of total SCFA, mainly due to acetate reduction, in the feces of dogs, especially dogs supplemented with scFOS. This is an interesting result, as acetate may be used as an energy source to produce glucose. Two studies in humans reported that patients with type-2 diabetes had a moderate degree of gut microbial dysbiosis, and a decreased amount of some universal butyrate-producing bacteria, in favor of propionate and acetate over butyrate levels [52,53]. An overproduction of SCFA has also been reported in obese individuals compared to normal subjects, notably due to a higher level of acetate, while the butyrate concentration was reduced [48]. Besides increasing microbial diversity, dietary supplementation with scFOS, by modulating the gut microbiota composition, might have alleviated such a shift in SCFA production.

Interestingly, scFOS supplementation significantly increased the composite score of the metavariable faecalGD2, characterized by phenylpropanoate. He et al. [54] reported an improvement in glucose homeostasis simultaneous with an increase in fecal phenylpropanoate in diabetic mice fed resistant maltodextrins. In addition, FaecalGD2 was negatively correlated to cholesterol, supporting the numerical decrease in the cholesterol level observed with scFOS supplementation. Taken together, these results suggest that scFOS allows for positive modulation of microbiota that may help improve glucose homeostasis and the lipid profile in obese dogs.

Overall, scFOS had a stronger effect on gut microbiota than OF, and the different effects are in favor of improving gut health and potentially improving the energy balance status in obese dogs, with a substantial numerical decrease in fasting insulin. We failed to demonstrate significant effects on glucose metabolism, probably due to the method we used but also to the low prebiotic dosage, the small number of animals, and the high inter-variability between dogs. The significant inter-individual variability in research dogs has already been described by Handl et al. [6]. Similar to us, they achieved a stronger individual effect than a diet effect. Indeed, from Table 4, it appears that, even if all dogs were overweighed, the range was important. In addition, certain dogs were insulin resistant while others were not. Due to this high inter-individual variability, we decided to build a correlations network between different parameters in order to understand the relationships between gut microbiota and host parameters and to see how prebiotics and/or proteins may modulate the energy balance in obese dogs.

The correlation network highlighted the functional block “GenusD1” corresponding to the combination of several bacterial genera. Its hub position in the network, connecting phenotypic (body weight), plasma (haptoglobin and cholesterol) and fecal metabolic/metabolomic features, and digestive endpoints (fecal parameters), effectively underlined the impact of the gut microbiota on host health.

Blood cholesterol, haptoglobin, fecal parameters, body weight, and excess body weight were linked to the metavariables GenusD1 and/or GenusD2. Furthermore, it underlined that fasting insulin and HOMA-IR were positively correlated with the metavariable GenusD3, and with excess body weight. These observations suggest that gut microbiota play a key role in the host energy balance in weight-disturbed dogs. More precisely, our results suggest that in obese dogs, there is a core of discriminant microbial populations, namely *Allobaculum, Lactobacillus, Bacteroides, Parabacteroides,* and *Gammaproteobacteria*, including *Escherichia-Schigella* and *Phascolarctobacterium*.

The genus *Allobaculum* was a discriminant genus in both metavariables GenusD1 and GenusD3, being associated with excess body weight and fasting insulin. Different studies using rodents showed that a low-fat diet was associated with an increase in the genus *Allobaculum* compared to a high-fat diet, or with excess body weight [46,55,56]. *Lactobacillus* was also reported as a discriminant genus in our study. There are discrepancies concerning the relationships between *Lactobacillus* and obesity, as certain authors reported an increase [57] while others a decrease [58] in this specific genus. The effects of *Lactobacillus* on obesity have been shown to be species and strain dependent and associated with either weight loss or weight gain.

The presence of *Gammaproteobacteria* unclassified and *Escherichia-Shigella* suggested that obese individuals were associated with a pro-inflammatory status. Identification of diaminoheptanedioate, a molecule in the cell wall of Gram-negative bacteria, as a discriminant metabolite found in the metavariable PlasmaLD2 suggested that obesity and its associated metabolic disorders are correlated to a high LPS level. Furthermore, hydroxyproline, a possible stress marker but also an obesity marker [59], was also a discriminant metabolite in our metavariable PlasmaLD2, while our metavariable GenusD1 including *Escherichia-Shigella* was positively correlated with blood haptoglobin, another inflammatory marker. These findings support the recent results obtained by Jergens et al. (2019; [10]) using both type 2 diabetes mellitus dogs and healthy dogs.

The presence of *Parabacteroides, Phascolarctobacterium*, and different *Bacteroides* might suggest a shift in SCFA production related to increased body weight. In a study using rats fed with a high-fat diet, these bacteria were positively correlated with a change in body weight [58]. *Phascolarctobacterium* spp. produce high amounts of acetate and propionate and are specialized in the utilization of succinate produced by other bacteria [60]. It is interesting to underline that this genus was associated with those of *Bacteroides* and *Parabacteroides*, two major succinate producers. Overall, these findings suggest that in obese dogs, microbiota are characterized by bacteria genera typical of a pro-inflammatory state and of modulation of the SCFA production pattern, as already suggested by Qin et al. [53]. To our knowledge, this is the first time that such observations have been made in the obese dog model.

The use of non-targeted metabolomics highlighted the importance of amino acid and bile acid metabolites. A recent study on humans with an impaired glucose tolerance similarly reported the importance of certain amino acids and bile acids [61]. Bennion and Grundy [62] previously observed that type-2 diabetic patients had an increased bile acid pool size and fecal excretion of bile acids. Other authors revealed that the bile acid pool composition was modified with type-2 diabetes, with notably higher microbial dihydroxylation of cholic acid to yield deoxycholic acid, while high concentrations of blood cholic and deoxycholic acids were associated with a higher risk of developing type-2 diabetes [61]. A recent study performed in dogs with or without diabetes mellitus demonstrated that the dogs with mellitus diabetes harbored more cholic acid in feces from mellitus diabetes-suffering dogs [10]. Our study confirmed the importance of bile acids for glucose homeostasis. However, we did not provide information about the bile acid concentrations in feces and did not focus on the relationships between bile acids and gut microbiota. Therefore, to further our understanding, we recommend measuring fecal bile acid concentrations along with microbiota analysis itself in lean and obese dogs. Indeed, if our observations are further validated, fecal bile acids could be interesting markers of glucose homeostasis failure in obese dogs.

Amino acids and microbial amino acid derivatives were also strongly present in our model. FaecalGD1 and PlasmaLD2 metavariables were indeed associated with phenylalanine, alanine, threonine, valine, lysine, leucine, and isoleucine or their derivatives. Blood branched-chain amino acid (BCAA) concentrations are known to be higher in obese humans and correlated with insulin levels [63,64]. Another recent study on humans with impaired glucose tolerance reported that phenylalanine, leucine, isoleucine, tyrosine, proline, and tyrosine increased the likelihood of developing type-2 diabetes [61]. Interestingly, in our study, the dietary protein level resulted in a trend (*p* = 0.09) modifying the metavariable PlasmaLD2, suggesting that a high-protein diet can modulate the different parameters of this metavariable directly in relation to fasting insulin.

The metavariable FaecalLD2 was positively correlated with blood cholesterol and FaecalGD2 while it was negatively correlated with FaecalGD1. FaecalLD2 was characterized by different types of molecules, namely hexanoylcarnitine and limonene, which were positively correlated to the metavariable, contrary to hippurate, acetylcholine, and aminoadipate, which were negatively correlated. Studies showed reduced hippurate excretion in obesity [65,66] while blood hippurate has been positively correlated with the Shannon diversity index, and a higher *Ruminococcaceae* and lower *Lachnospiraceae* abundance [67]. Aminoadipate is generated by lysine degradation and may also serve as a substrate for enzymes downstream of tryptophan metabolism. A study reported that individuals from two independent cohorts exhibiting high plasma aminoadipate concentrations had up to a 4-fold risk of developing diabetes in the future [68]. The authors suggested plasma aminoadipate as a potential marker for diabetes risk. Our study suggested that in obese dogs, hippurate and aminoadipate were two discriminant metabolites in fecal water and could be useful markers for predicting insulin-resistance risk. Further research, including controls and insulin-resistant dogs to measure precise fecal concentrations, should be developed to confirm these findings.

## 5. Conclusions

Our study highlighted that, even if glucose homeostasis was not significantly modified, dietary supplementation with scFOS modulated the gut microbiota composition and activity (especially the increase in butyrate and the decrease in total SCFAs) in a way that seemed, even if not significant, beneficial for glucose homeostasis in obese dogs. This work also effectively illustrated the usefulness of analyzing heterogeneous data stratified in “functional” latent variables (or block) to display as a network the complex relationship between the gut microbiota and the host. Such an approach has already demonstrated its efficiency in other fields of health [69,70].

The correlations network showed a core of bacteria directly related to excess body weight and glucose homeostasis in obese dogs. In particular, we found that *Gammaproteobacteria* unclassified and *Escherishia-Shigella* were present as discriminant bacteria in all our metavariables. These bacteria are linked to a pro-inflammatory state, and the presence of diaminoheptanedioate as a discriminant metabolite in plasma is consistent with this. The presence of *Parabacteroides, Bacteroides* unclassified, and *Phascolarctobacterium* in the same metavariable suggests a potential shift in SCFA production, especially a higher production of acetate and propionate, using succinate as substrate in dogs that are the most overweight. Such findings may be useful for developing nutritional strategies targeted against relevant species linked to changes in body weight or glucose homeostasis failure in obese dogs. Furthermore, we confirmed the importance of certain amino acids, especially phenylalanine and its derivatives, and of bile acids in glucose homeostasis. In addition, we suggested fecal hippurate and aminoadipate as potential markers of glucose homeostasis. To consolidate these preliminary results, further investigations using more dogs, with a control and an obese group, and determining fecal hippurate, aminoadipate, and bile acid concentrations are needed. Overall, further work is required to find relevant non-invasive markers to evaluate both glucose homeostasis and the effects of the nutritional strategies used.

## Figures and Tables

**Table 1:** Analyzed composition of the basal diets as fed to dogs.

Ingredients	Normal Protein	High Protein
	**g/kg**	**g/kg**
Cereals and derivatives	595.6	522.0
Meat and derivatives	350.0	423.6
Beet pulp	20	20
Hydrolysates	20	20
Linseed	10	10
Mineral Premix	3.75	3.74
Calcium propionate	0.5	0.5
Vitamin premix	0.15	0.15
Dry matter	884	894
	**g/100 g DM**	**g/100 g DM**
Protein	25.6	36.9
Fat	11.6	11.7
Ash	9.4	9.5
NFE	47.8	36.8
Starch	34.1	26.1
Crude fiber (ADF)	5.6	5.1
Calcium	2.5	2.5
Phosphorus	1.5	1.6

DM: dry matter. Manufacturer: SOPRAL.

**Table 2:** Averaged effects of protein level and prebiotic supplementation on feces characteristics and fecal concentrations of fermentation end-products.

	Protein		Prebiotic			*p*-Value	
	Normal	High	CTRL	OF	scFOS	Protein	Prebiotic
Body weight, kg	13.5 ± 0.31	13.4 ± 0.31	13.5 ± 0.47	13.3 ± 0.43	13.4 ± 0.34	0.664	0.917
Excess body weight, %	51.6 ± 3.40	52.9 ± 6.13	54.4 ± 7.08	51.8 ± 4.98	50.1 ± 5.50	0.875	0.149
Fasting glucose, mmol/L	5.29 ± 0.07	5.17 ± 0.06	5.21 ± 0.09	5.32 ± 0.08	5.16 ± 0.06	0.292	0.418
Fasting insulin, mU/L	17.1 ± 2.07	15.7 ± 1.99	17.0 ± 2.85	17.6 ± 2.42	14.6 ± 2.15	0.628	0.613
Haptoglobin, g/L	2.01 ± 0.18	2.10 ± 0.41	1.99 ± 0.25	2.44 ± 0.54	1.70 ± 0.21	0.832	0.499
Cholesterol, mmol/L	7.4 ± 0.27	7.1 ± 0.33	7.5 ± 0.39	7.2 ± 0.37	7.0 ± 0.33	0.292	0.727

Data are given as means ± SEM.

**Table 3:** Averaged effects of protein level and prebiotic supplementation on feces characteristics and fecal concentrations of fermentation end-products.

	Protein		Prebiotic			*p*-Value	
	Normal	High	CTRL	OF	scFOS	Protein	Prebiotic
Total fecal output, g/d	573 ± 31.6	503 ± 25.7	544 ± 35.4	516 ± 31	563 ± 46.8	0.548	0.841
Fecal dry matter, %	40.3 ± 0.98	41.7 ± 1.08	41.4 ± 1.03	41.3 ± 1.58	40.2 ± 1.15	0.319	0.894
Stool frequency/d	2.58 ± 0.156	2.17 ± 0.103	2.36 ± 0.176	2.33 ± 0.167	2.48 ± 0.198	0.380	0.933
**Volatile fatty acids (VFA), µg/mL fecal water**							
Butyrate	717 ± 84.5	841 ± 101.4	713 ^a^ ± 74.1	728 ^a^ ± 106.2	895 ^b^ ± 155.2	0.636	0.039
Acetate	3383 ± 322	3963 ± 337	4096 ± 253	3265 ± 338	3598 ± 589	0.546	0.646
Propionate	1736 ± 251	2352 ± 334	2059 ± 119	1857 ± 323	2172 ± 578	0.244	0.880
Total VFA	5836 ± 610	7155 ± 740	6868 ± 406	5849 ± 725	6665 ± 1281	0.479	*0.109*
**Branched-chain fatty acids (BCFA), µg/mL fecal water**							
Isobutyrate	139 ± 14.3	176 ± 15.9	190 ± 13.7	135 ± 18.2	142 ± 21.9	0.311	0.306
Isovalerate	190 ± 20.0	237 ± 19.1	260 ± 19.9	181 ± 22.0	194 ± 27.4	0.307	0.249
Valerate	19.0 ± 4.01	20.1 ± 1.94	17.8 ± 1.86	21.3 ± 4.60	19.5 ± 5.17	0.952	0.875
Total BCFA	348 ± 35.9	433 ± 35.8	468 ± 34.3	337 ± 41.5	356 ± 52.7	0.340	0.328

a, b: Means within a raw with different superscripts differ significantly. Data are given as means ± SEM.

**Table 4:** Characteristics of the phenotypic data used in the correlations network.

Item	*n*	Mean	Median	Min	Max	SD
Body weight, kg	36	13.56	13.75	10.55	15.70	1.36
Excess body weight, %	36	53.51	53.00	19.90	90.00	18.45
Fasting glucose, mmol/L	36	5.24	5.30	4.60	5.90	0.262
Fasting insulin, mU/L	36	16.64	13.00	7.00	40.00	8.60
HOMA-IR	36	2.12	1.63	0.92	4.98	1.16
Haptoglobin, mg/L	36	2140	1743	949	7268	1335
Cholesterol, mmol/L	36	7.35	7.47	4.87	9.59	1.19
Total fecal output, g/d	36	540.8	536.1	311.5	786.0	121.3
Fecal dry matter, %	36	41.1	41.5	34.2	50.1	4.00
Stool frequency, /day	36	2.35	2.40	1.40	3.80	0.59

**Table 5:** Metabolites, their main metabolic pathways, and the correlation coefficient between each metabolite and the concerned metavariable.

Metabolite	Metabolic Pathways	Correlation Coefficient
**Fecal water LD1**		
Cholic acids	Primary bile acids	0.89 0.88 0.87 0.86
Deoxycholic acids	Secondary bile acids	0.87 0.78
Taurocholic acids	Primary bile acids	0.80 0.76
L-Phenylalanine	Amino acid	0.85
Norleucine	Derived from lysine, involved in lipid metabolism	0.82
Tryptophan	Amino acid	0.82
**Fecal water GD1**		
L-Alanine	Amino acid	0.91
L-Valine	Amino acid	0.90
Cadaverine	Lysine metabolism	0.89
L-Proline	Amino acid	0.87
L-Threonine	Amino acid	0.86
L-Phenylalanine	Amino acid	0.86
**Fecal water LD2**		
Hexanoylcarnitine	Lipid and amino-acid metabolism	0.67
Limonene	Lipid metabolism connected with primary bile acid metabolism	0.65
Hippurate	Phenylalanine metabolism	−0.37
Aminoadipate	Lysine metabolism	−0.52
Acetylcholine	Neurotransmitter involved in many functions including insulin, bile and pancreatic secretion	−0.63
**Fecal water GD2**		
Phenylpropanoate	Aromatic compounds, phenylalanine degradation, polyphenol metabolism	0.62
D-Fructose	Amino sugar and nucleotide sugar metabolism	0.55
**PlasmaLD2**		
Leucine Aspargine	Dipeptide – Amino-acid/protein metabolism	0.74
Cis or trans-4- Hydroxy-D-proline	Proline and derivatives – Marker of bone resorption, muscle degradation, depression and stress	0.72
Diaminoheptanedioate	Derived from lysine, specific to certain cell walls of gram-negative bacteria	0.69
Acylcarnitine	Produced from lysine and methionine, involved in fatty acid catabolism	−0.46

**Table 6:** Averaged means of microbial and metabolomic metavariables according to protein level and prebiotic supplementation.

	Protein		Prebiotic			*p*-Value	
	Normal	High	CTRL	OF	scFOS	Protein	Prebiotic
**Plasma metabolome**							
PlasmaLD2	0.29 ± 41	−0.33 ± 0.47	−0.19 ± 0.55	−0.16 ± 0.57	0.38	0.099	0.892
**Fecal metabolome**							
FaecalGD1	−0.11 ± 0.89	0.13 ± 0.83	−0.36 ± 0.82	−0.17 ± 1.10	0.57 ± 1.28	0.910	0.937
FaecalGD2*	−0.22 ± 0.62	0.24 ± 0.55	0.98 ± 0.80	0.92 ± 0.61	2.88 ± 0.67	0.609	0.013
FaecalLD1	0.72 ± 0.91	−0.82 ± 0.96	−1.73 ± 0.50	0.49 ± 1.33	1.37 ± 1.35	0.121	0.372
FaecalLD2	0.51 ± 0.46	−0.58 ± 0.58	−0.04 ± 0.56	0.20 ± 0.71	−0.18 ± 0.70	0.420	0.741
**Metagenome**							
GenusD1	−0.71 ± 0.55	0.81 ± 0.46	0.80 ± 0.72	−0.11 ± 0.76	−0.77 ± 0.37	0.367	0.429
GenusD2	0.17 ± 0.49	−0.19 ± 0.48	0.17 ± 0.68	0.13 ± 0.60	−0.33 ± 0.48	0.598	0.935
GenusD3	−0.02 ± 0.51	0.02 ± 0.38	−0.56 ± 0.45	0.66 ± 0.74	−0.11 ± 0.14	0.921	0.601

Data are given as means ± SEM.

**Table 7:** Main metabolites associated with each metavariable and correlation between each metabolite and the metavariable.

Bacterial Genus	Correlation Coefficient with the Metavariables
*GenusD1*	
*Lactobacillus*	0.75
*Bacteroides unclassified*	0.61
*Allobaculum*	0.60
*Lactobacillales unclassified*	0.54
*Escherichia Shigella*	0.52
*Parabacteroides*	0.48
*Campylobacter*	−0.49
***GenusD2***	
*Parabacteroides*	0.61
*Phascolarctobacterium*	0.57
*Campylobacter*	0.51
*Gammaproteobacteria unclassified*	0.49
*Fusobacterium*	−0.48
*Wolinella*	−0.51
*Brachyspira*	−0.59
***GenusD3***	
*Enterococcus*	0.67
*Escherichia Shigella*	0.59
*Allobaculum*	0.48
*Allisonella*	0.45
*Campylobacter*	0.44
*Clostridium sensu stricto1*	−0.43
*Anaeroplasma*	−0.55

**Table 8:** Partial correlation coefficient, *p*-value, and Q-value obtained between different variables and metavariables in the correlations network.

Node 1	Node 2	Partial Correlation Coefficient	*p*-Value	Q-value
HOMA IR	Fasting insulin	0.415	4.44 × 10^−16^	3.03 × 10^−14^
FaecalLD1	FaecalGD1	0.316	1.17 × 10^−9^	4.32 × 10^−8^
FaecalLD2	FaecalGD2	0.315	1.37 × 10^−9^	4.68 × 10^−8^
Body weight	Excess body weight %	0.298	1.06 × 10^−8^	2.90 × 10^−7^
Haptoglobin	Cholesterol	−0.237	6.42 × 10^−6^	0.0001
GenusD2	Body weight	0.219	3.27 × 10^−5^	0.0006
PlasmaLD2	FaecalLD1	0.206	9.00 × 10^−5^	0.0014
GenusD1	Excess body weight %	9.05 × 10^−5^	0.206	0.0014
Total fecal output	Stool frequency	0.198	0.0002	0.0024
Haptoglobin	Fecal dry matter %	0.194	0.0002	0.0030
FaecalLD2	FaecalGD1	−0.179	0.0007	0.0082
GenusD1	Cholesterol	0.160	0.0025	0.0222
Fecal dry matter %	Stool frequency	−0.155	0.0034	0.0273
Total fecal output	Fecal dry matter %	−0.154	0.0036	0.0287
GenusD1	Fecal dry matter %	0.152	0.0040	0.0305
FaecalLD2	Cholesterol	0.152	0.0040	0.0305
GenusD1	Haptoglobin	0.148	0.0053	0.0371
GenusD2	Stool frequency	−0.147	0.0057	0.0389
PlasmaLD2	Haptoglobin	−0.136	0.0104	0.0618
FaecalLD1	Fasting glucose	0.136	0.0105	0.0618
PlasmaLD2	HOMA IR	0.133	0.0123	0.0702
PlasmaLD2	Fecal dry matter %	0.125	0.0190	0.0956
PlasmaLD2	Fasting insulin	0.123	0.0207	0.1013
HOMA IR	Excess body weight %	0.122	0.0214	0.1035
FaecalGD1	Stool frequency	0.116	0.0290	0.1247
GenusD3	HOMA IR	0.115	0.0301	0.1272
FaecalGD2	Cholesterol	−0.115	0.0310	0.1296
FaecalLD2	GenusD1	−0.113	0.0337	0.1356
GenusD3	Fasting insulin	0.111	0.0359	0.1402

## References

[1] Toll P., Yamka R.M., Schoenherr W.D., Hand M.S., Thatcher C.D., Remillard R.L., Roudebush P., Norvotny B.J. (2010). Obesity. Small Animal Clinical Nutrition.

[2] Colliard L., Ancel J., Benet J.J., Paragon B.M., Blanchard G. (2006). Risk factors for obesity in dogs in France. J. Nutr..

[3] Holmes K.L., Morris P.J., Abdulla Z., Hackett R., Rawlings J.M. (2007). Risk factors associated with excess body weight in dogs in the UK. J. Anim. Physiol. Anim. Nutr..

[4] Tvarijonaviciute A., Ceron J.J., Holden S.L., Cuthbertson D.J., Biourge V., Morris P.J., German A.J. (2012). Obesity-related metabolic dysfunction in dogs: A comparison with human metabolic syndrome. BMC Vet. Res..

[5] Cani P.D., Amar J., Iglesias M.A., Poggi M., Knauf C., Bastelica D., Neyrinck A.M., Fava F., Tuohy K.M., Chabo C.W. (2007). Metabolic endotoxemia initiates obesity and insulin resistance. Diabetes.

[6] Handl S., German A.J., Holden S.L., Dowd S.E., Steiner J.M., Heilmann R.M., Grant R.W., Swanson K.S., Suchodolski J.S. (2013). Faecal microbiota in lean and obese dogs. FEMS Microbiol. Ecol..

[7] Rahat-Rozenbloom S., Fernandes J., Gloor G.B., Wolever T.M. (2014). Evidence for greater production of colonic short-chain fatty acids in overweight than lean humans. Int. J. Obes. (Lond.).

[8] McNeil N.I. (1984). The contribution of the large intestine to energy supplies in man. Am. J. Clin. Nutr..

[9] Park H.J., Lee S.E., Kim H.B., Isaacson R.E., Seo K.W., Song K.H. (2015). Association of obesity with serum leptin, adiponectin, and serotonin and gut microflora in beagle dogs. J. Vet. Intern. Med..

[10] Jergens A.E., Guard B.C., Redfern A., Rossi G., Mochel J.P., Pilla R., Chandra L., Seo Y.J., Steiner J.M., Lidbury J. (2019). Microbiota-related changes in unconjugated fecal bile acids are associated with naturally occurring, insulin-dependent diabetes mellitus in dogs. Front. Vet. Sci..

[11] Allin K.H., Nielsen T., Pedersen O. (2015). Mechanisms in endocrinology: Gut microbiota in patients with type 2 diabetes mellitus. Eur. J. Endocrinol..

[12] Diez M., Nguyen P., Jeusette I., Devois C., Istasse L., Biourge V. (2002). Weight loss in obese dogs: Evaluation of a high-protein, low-carbohydrate diet. J. Nutr..

[13] German A.J., Holden S.L., Bissot T., Morris P.J., Biourge V. (2012). A high protein high fibre diet improves weight loss in obese dogs. Vet. J..

[14] André A., Leriche I., Chaix G., Thorin C., Burger M., Nguyen P. (2017). Recovery of insulin sensitivity and optimal body composition after rapid weight loss in obese dogs fed a high-protein medium-carbohydrate diet. J. Anim. Physiol. Anim. Nutr..

[15] Chevrier G., Mitchell P., Beaudoin M.S., Marette A. (2016). Impact of dietary proteins on energy balance, insulin sensitivity and glucose homeostasis: From proteins to peptides to amino acids. the Molecular Nutrition of Amino Acids and Proteins.

[16] Pais R., Gribble F.M., Reimann F. (2016). Stimulation of incretin secreting cells. Ther. Adv. Endocrinol. Metab..

[17] Lubbs D.C., Vester B.M., Fastinger N.D., Swanson K.S. (2009). Dietary protein concentration affects intestinal microbiota of adult cats: A study using DGGE and qPCR to evaluate differences in microbial populations in the feline gastrointestinal tract. J. Anim. Physiol. Anim. Nutr..

[18] Gibson G.R., Scott K.P., Rastall R.A., Tuohy K.M., Hotchkiss A., Dubert-Ferrandon A., Gareau M., Murphy E.F., Saulnier D., Loh G. (2010). Dietary prebiotics: Current status and new definition. Food Sci. Technol..

[19] Swanson K., Grieshop C., Flickinger E., Bauer L., Chow J., Wolf B., Garleb K., Fahey G. (2002). Fructo-oligosaccharides and *Lactobacillus acidophilus* modify gut microbial populations, total tract digestibilities and fecal protein catabolite in healthy adult dogs. J. Nutr..

[20] Respondek F., Swanson K.S., Belsito K.R., Vester B.M., Wagner A., Istasse L., Diez M. (2008). Short-chain fructo-oligosaccharides influence insulin sensitivity and gene expression of fat tissue in obese dogs. J. Nutr..

[21] Massimino S.P., McBurney M.I., Field C.J., Thomson A.B., Keelan M., Hayek M.G., Sunvold G.D. (1998). Fermentable dietary fiber increases GLP-1 secretion and improves glucose homeostasis despite increased intestinal glucose transport capacity in healthy dogs. J. Nutr..

[22] Daumas C., Lhoest E., Hornick J.L., Istasse L., Diez M. Development of a practical test of insulin resistance in obese Beagle dogs and effects of scFOS. Proceedings of the 13th Congress of the European Society of Veterinary and Comparative Nutrition.

[23] Laflamme D.P. (1997). Development and validation of a body condition score system for dogs. Canine Pract..

[24] National Research Council (1985). Nutrient Requirements of Dogs.

[25] (2016). WALTHAM. https://www.waltham.com.

[26] Verkest K.R., Fleeman L.M., Rand J.S., Morton J.M. (2010). Basal measures of insulin sensitivity and insulin secretion simplified glucose tolerance tests in dogs. Domest. Anim. Endocrinol..

[27] Humblet M., Coghe J., Lekeux P., Godeau J.M. (2004). Acute phase proteins assessment for an early selection of treatments in growing calves suffering from bronchopneumonia under field conditions. Res. Vet. Sci..

[28] Quast C., Pruesse E., Yilmaz P., Gerken J., Schweer T., Yarza T., Peplies P., Glockner F.O. (2012). The SILVA ribosomal RNA gene database project: Improved data processing and web-based tools. Nucleic Acids Res..

[29] Hunter P.R., Gaston M.A. (1988). Numerical index of the discriminatory ability of typing systems: An application of Simpson’s index of diversity. J. Clin. Microbiol..

[30] Bray J.R., Curtis J.T. (1957). An ordination of upland forest communities of southern Wisconsin. Ecol. Monogr..

[31] Martin A.P. (2002). Phylogenetic approaches for describing and comparing the diversity of microbial communities. Appl. Environ. Microbiol..

[32] Parks D.H., Beiko R. (2010). GIdentifying biologically relevant differences between metagenomic communities. Bioinformatics.

[33] Opgen-Rhein R., Strimmer K. (2006). Inferring gene dependency networks from genomic longitudinal data: A functional data approach. Rev. Stat..

[34] Opgen-Rhein R., Strimmer K. Using regularized dynamic correlation to infer gene dependency networks from time-series microarray data. Proceedings of the 4th International Workshop on Computational Systems Biology.

[35] Schäfer J., Strimmer K. (2005). A shrinkage approach to large-scale covariance matrix estimation and implications for functional genomics. Stat. Appl. Genet. Mol. Biol..

[36] Jeusette I., Grauwels M., Cuvelier C., Tonglet C., Istasse L., Diez M. (2004). Hypercholesterolaemia in a family of rough collie dogs. J. Small Anim. Pract..

[37] Ricci R., Jeusette I., Godeau J.M., Contiero B., Diez M. (2011). Effect of short-chain fructo-oligosaccharide-enriched energy-restricted diet on weight loss and serum haptoglobin concentration in Beagle dogs. Br. J. Nutr..

[38] Verbrugghe A., Hesta M., Gommeren K., Daminet S., Wuyts B., Buyse J., Janssens G.P. (2009). Oligofructose and inulin modulate glucose and amino acid metabolism through propionate production in normal-weight and obese cats. Br. J. Nutr..

[39] Alexander C., Liu T.W., Devendran S., Theis S., Ridlon J.M., Suchodolski J.S., de Godoy M.R.C., Swanson K.S. (2017). Effects of prebiotic inulin-type fructans on blood metabolite and hormone concentrations and fecal microbiota and bile acids in overweight dogs. FASEB J..

[40] Le Bourgot C., Apper E., Blat S., Respondek F. (2018). Fructo-oligosaccharides and glucose homeostasis: A systematic review and meta-analysis in animal models. Nutr. Metabol..

[41] Kellow N.J., Coughlan M.T., Reid C.M. (2014). Metabolic benefits of dietary prebiotics in human subjects: A systematic review of randomised controlled trials. Br. J. Nutr..

[42] Ader M., Stefanovski D., Richey J.M., Kim S.P., Kolka C.M., Ionut K.J., Catalano K., Hucking M., Ellmerer G., Van Citters I.R. (2014). Failure of homeostatic model assessment of insulin resistance to detect marked diet-induced insulin resistance in dogs. Diabetes.

[43] Ley R.E., Backhed F., Turnbaugh P., Lozupone C.A., Knight R.D., Gordon J.I. (2005). Obesity alters gut microbial ecology. Proc. Natl. Acad. Sci. USA.

[44] Turnbaugh P.J., Hamady M., Yatsunenko T., Cantarel B.L., Duncan A., Ley R.E., Sogin M.L., Jones W.J., Roe B.A., Affourtit J.P. (2009). A core gut microbiome in obese and lean twins. Nature.

[45] Menni C., Jackson M.A., Pallister T., Steves C.J., Spector T.D., Valdes A.M. (2017). Gut microbiome diversity and high-fibre intake are related to lower long-term weight gain. Int. J. Obes..

[46] Ravussin Y., Koren O., Spor A., LeDuc C., Gutman R., Stombaugh J., Knight R., Ley R.E., Leibel R.L. (2012). Responses of gut microbiota to diet composition and weight loss in lean and obese mice. Obesity.

[47] Zeber-Lubecka N., Kulecka M., Ambrozkiewicz F., Paziewska A., Goryca K., Karczmarski J., Rubel T., Wojtowicz W., Mlynarz P., Tomecki R. (2016). Limited prolonged effects of rifaximin treatment on irritable bowel syndrome-related differences in the fecal microbiome and metabolome. Gut Microbes.

[48] Patil D.P., Dhotre D.P., Chavan S.G., Sultan A., Jain D.S., Lanjekar V.B., Gangawani J., Shah P.S., Todkar J.S., Shah S. (2012). Molecular analysis of gut microbiota in obesity among Indian individuals. J. Biosci..

[49] Collado M.C., Isolauri E., Laitinen K., Salminen S. (2008). Distinct composition of gut microbiota during pregnancy in overweight and normal-weight women. Am. J. Clin. Nutr..

[50] Roesch L.F., Lorca G.L., Casella G., Giongo A., Naranjo A., Pionzio A.M., Li N., Mai V., Wasserfall C.H., Schatz D. (2009). Culture-independent identification of gut bacteria correlated with the onset of diabetes in a rat model. ISME J..

[51] Peng L., Li Z.R., Green R.S., Holzman I.R., Lin J. (2009). Butyrate enhances the intestinal barrier by facilitating tight junction assembly via activation of AMP-activated protein kinase in Caco-2 cell monoplayers. J. Nutr..

[52] Schwiertz A., Taras D., Schäfer K., Beijer S., Bos N.A., Donus C., Hardt P.D. (2010). Microbiota and SCFA in lean and overweight healthy subjects. Obesity.

[53] Qin J., Li Y., Cai Z., Li S., Zhu J., Zhang F., Liang S., Zhang W., Guan Y., Wang J. (2012). A metagenome-wide association study of gut microbiota in type 2 diabetes. Nature.

[54] He B., Nohara K., Ajami N., Michalek R.D., Tian X., Wong M., Losee-Olson S.H., Petrosino J.F., Yoo S.H., Chen Z. (2015). Transmissible microbial and metabolomic remodeling by soluble dietary fiber improves metabolic homeostasis. Sci. Rep. UK.

[55] Everard A., Lazarevic V., Gaïa N., Johansson M., Ståhlman M., Backhed F., Delzenne N.M., Schrenzel J., François P., Cani P.D. (2014). Microbiome of prebiotic-treated mice reveals novel targets involved in host response during obesity. ISME J..

[56] Kong C., Gao R., Yan X., Huang L., Qin H. (2019). Probiotics improve gut microbiota dysbiosis in obese mice fed a high-fat or high-sucrose diet. Nutrition.

[57] Million M., Angelakis E., Paul M., Armougom F., Leibovici L., Raoult D. (2012). Comparative meta-analysis of the effect of Lactobacillus species on weight gain in humans and animals. Microb. Pathog..

[58] Lecomte V., Kaakoush N.O., Maloney C.A., Raipuria M., Huinao K.D., Mitchell H.M., Morris M.J. (2015). Changes in gut microbiota in rats fed a high fat diet correlate with obesity-associated metabolic parameters. PLoS ONE.

[59] Goodson J.M., Hardt M., Hartman M., Schulte F., Tavares M., Mutawa A., Ariga J., Soparkar P., Behbehani J., Behbehani K. (2018). Identification of salivary and plasma biomarkers for obesity in children by non-targeted metabolomic analysis. BioRxiv.

[60] Watanabe Y., Nagai F., Morotomi M. (2011). Characterization of *Phascolarctobacterium succinatutens* sp. nov., an asaccharolytic, succinate-utilizing bacterium isolated from human feces. Appl. Environ. Microbiol..

[61] De Mello V.D., Paananen J., Lindström J., Lankinen M.A., Shi L., Kuusisto J., Pihlajamäki J., Auriola S., Lehtonen M., Rolandsson O. (2017). Indolepropionic acid and novel lipid metabolites are associated with a lower risk of type 2 diabetes in the Finnish Diabetes Prevention Study. Sci. Rep.UK.

[62] Bennion L.J., Grundy S.M. (1977). Effects of diabetes mellitus on cholesterol metabolism in man. N. Engl. J. Med..

[63] Felig P., Marliss E., Cahill G.F. (1969). Plasma amino acid levels and insulin secretion in obesity. N. Engl. J. Med..

[64] Newgard C.B., An J., Bain J.R., Muehlbauer M.J., Stevens R.D., Lien L.F., Haqq M.A., Shah S.H., Arlotto M., Slentz C.A. (2009). A branched-chain amino acid-related metabolic signature that differentiates obese and lean humans and contributes to insulin resistance. Cell Metab..

[65] Calvani R., Miccheli A., Capuani G., Tomassini Miccheli A., Puccetti C., Delfini M., Iaconelli A., Nanni G., Mingrone G. (2005). Gut microbiome-derived metabolites characterize a peculiar obese urinary metabotype. Int. J. Obes..

[66] Shearer J., Duggan G., Weljie A., Hittel D.S., Wasserman D.H., Vogel H.J. (2008). Metabolomic profiling of dietary-induced insulin resistance in the high fat-fed C57BL/6J mouse. Diabetes Obes. Metab..

[67] Pallister T., Jackson M.A., Martin T.C., Zierer J., Jennings A., Mohney R.P., MacGregor A., Steves C.J., Spector T.D., Menni C. (2017). Hippurate as a metabolomic marker of gut microbiome diversity: Modulation by diet and relationship to metabolic syndrome. Sci. Rep. UK.

[68] Wang T.J., Ngo D., Psychogios N., Dejam A., Larson M.G., Vasan R.S., Ghorbani A., O’Sullivan J., Cheng S., Rhee E.P. (2013). 2-Aminoadipic acid is a biomarker for diabetes risk. J. Clin. Investig..

[69] Wahl S., Vogt S., Stuckler F., Krumsiek J., Bartel J., Kacprowski T., Schramm K., Carstensen M., Rathmann W., Roden M. (2015). Multi-omic signature of body weight change: Results from a population-based cohort study. BMC Med..

[70] Martin J.C., Berton A., Ginies C., Bott R., Scheercousse P., Saddi A., Gripois D., Landrier J.F., Dalemans D., Alessi M.C. (2015). Multi-level systems biology modeling characterized the atheroprotective efficiencies of modified dairy fats in a hamster model. Am. J. Physiol. Heart Circ. Physiol..

